# Successful transcatheter arterial embolization for a massive hemothorax caused by acupuncture^☆^

**DOI:** 10.1016/j.radcr.2022.05.040

**Published:** 2022-06-24

**Authors:** Yui Hanabusa, Takatoshi Kubo, Takeyuki Watadani, Masaaki Nagano, Jun Nakajima, Osamu Abe

**Affiliations:** a Department of Radiology, The University of Tokyo Hospital, 7-3-1 Hongo, Bunkyo-ku, Tokyo, 113-8655, Japan; b Department of Thoracic Surgery, The University of Tokyo Hospital, 7-3-1 Hongo, Bunkyo-ku, Tokyo, 113-8655, Japan

**Keywords:** Transcatheter arterial embolization, Hemothorax, Acupuncture

## Abstract

Acupuncture is an alternative treatment for a variety of diseases, and serious complications are rare. We report a case of transcatheter arterial embolization performed in a patient with a massive hemothorax after acupuncture treatment.

A 36-year-old woman with no previous medical history was admitted to our hospital with left back pain and respiratory distress after acupuncture treatment. Contrast-enhanced computed tomography showed a left hemothorax and leakage of contrast medium, which was considered to result from an injury to the second intercostal artery, caused by acupuncture treatment. Transcatheter arterial embolization successfully stopped the bleeding, and the hematoma was thoracoscopically removed. No rebleeding was observed 6 months after treatment.

## Introduction

Acupuncture is one of the oldest alternative treatments for various diseases and conditions, and it has recently gained increased attention in Western countries [1]. Acupuncture involves the insertion and manipulation of fine needles to provide weak electrical or thermal stimulations to points related to the disease or condition being treated. Serious adverse events associated with acupuncture are rare; however, pneumothorax, vascular injury, central and peripheral nerve injury, organ injury, and infection have been reported [2]. There have been no reports of transcatheter arterial embolization (TAE) being used for the treatment of acupuncture-induced hemothorax. In this report, we present a case of TAE for a massive hemothorax after acupuncture.

## Case report

**Fig. 1:**
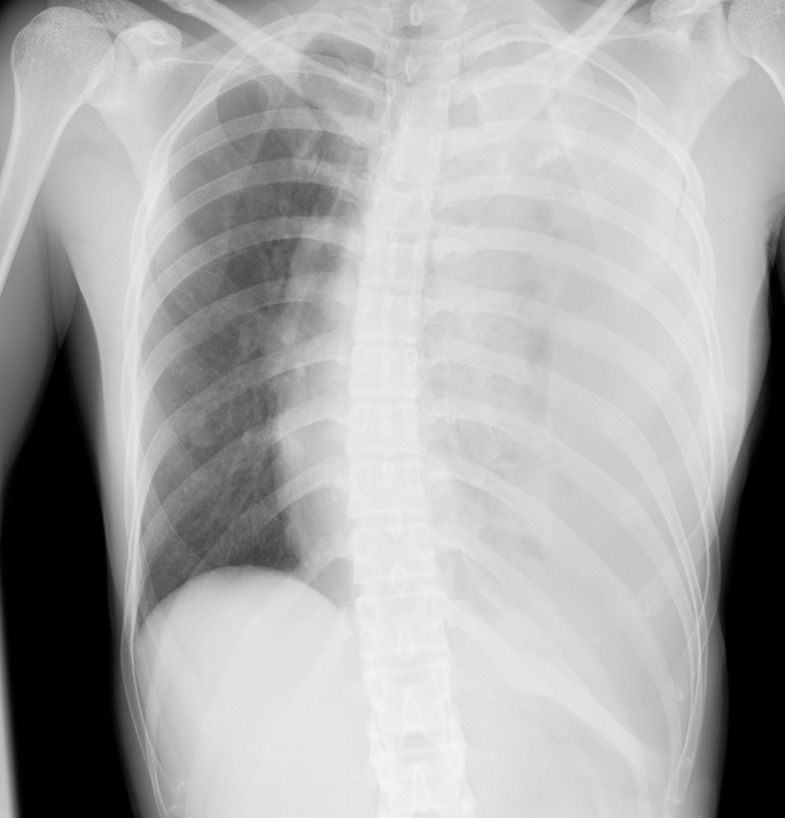
Chest radiograph on admission. Chest radiograph showing a massive left pleural effusion, with mediastinal deviation to the right.

**Fig. 2:**
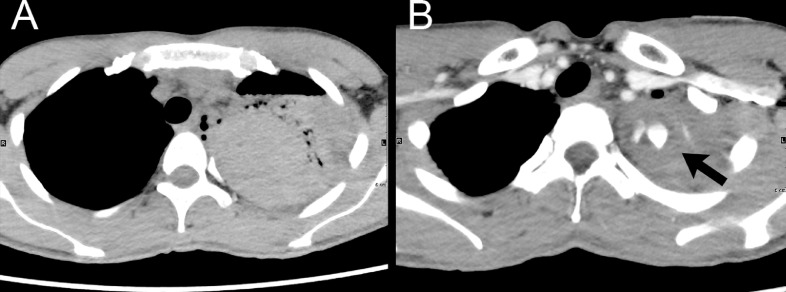
Computed tomography (CT) before transcatheter arterial embolization. (A) Left massive hemothorax was observed on CT. (B) Contrast leakage into the left thoracic cavity indicated active bleeding (arrow).

**Fig. 3:**
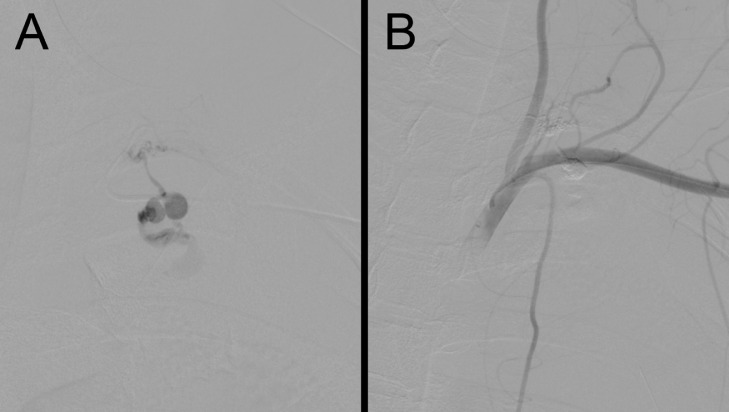
Digital subtraction angiography (DSA) of transcatheter arterial embolization. (A) DSA of the left second intercostal artery showing active bleeding. (B) No residual bleeding was observed after embolization.

A 36-year-old woman with no prior medical history or trauma received acupuncture therapy to the left back for muscle pain. After treatment, left back pain appeared and gradually worsened. She presented with intense exacerbation of pain, dyspnea, and nausea the next morning and was admitted to our hospital 14 hours after acupuncture. On admission, her pulse rate was 150/min, her blood pressure was 88/54 mm Hg, her respiration rate was 24/min, and her oxygen saturation was 88% (with an 8 L/min oxygen supply). Respiratory sounds were diminished on the left side, and a chest radiograph showed a massive left pleural effusion, with mediastinal deviation to the right (Fig. 1). Drainage of the left thoracic cavity was performed, with initial drainage of approximately 300 mL of bloody pleural effusion, followed by a 1000-mL drainage 1.5 hours later. Laboratory results showed anemia, with a hemoglobin level of 9.8 mg/dL, which reduced to 6.4 mg/dL 2 hours later. Contrast-enhanced computed tomography (CT) showed a massive left hemothorax with contrast leakage into the left thoracic cavity (Fig. 2); thus, emergency TAE was planned. A sheath was inserted through the left brachial artery, and a 4-Fr catheter (Seiha, MEDIKIT, Tokyo, Japan) was advanced into the left subclavian artery. Digital subtraction angiography (DSA) revealed a pseudoaneurysm in the second intercostal artery. A 2.1-Fr microcatheter (Sniper 2 Selective, TERUMO, Tokyo, Japan) was inserted into the second intercostal artery via the costocervical artery; DSA confirmed active bleeding (Fig. 3A). A 33% n-butyl cyanoacrylate-lipiodol mixture was injected into the second intercostal artery, and embolization was achieved across the affected area. DSA after embolization confirmed the disappearance of bleeding and absence of non-targeted embolization (Fig. 3B).

After TAE, the patient underwent thoracoscopic hematoma removal because of poor drainage of the left intrathoracic hematoma. Intraoperative findings revealed no residual bleeding, left lung injury, bullae, or menstrual pneumothorax. The patient had good postoperative lung expansion and was discharged one week after TAE. Contrast-enhanced CT 6 months later showed no rebleeding.

## Discussion

Acupuncture is an alternative treatment employed for several purposes, including pain relief, and its modalities have been increasing in recent years, mainly in Western countries [1]. However, with an increase in the number of acupuncture modalities comes an increase in adverse event reports related to acupuncture [2], [3], [4], [5].

The frequency of serious adverse events varies among the reports. A systematic review by Wu et al. [2] reported 182 serious adverse events (including 24 deaths) in China over 33 years, from 1980 to 2013. These include pneumothorax, central and peripheral nerve injury, organ injury, infection, bleeding, and residual broken needles, among others. Endres et al. [3] reported that of 190,924 patients that underwent acupuncture, 14,404 (7.5%) patients experienced adverse events, but only 45 (0.02%) patients experienced serious adverse events. However, Witt et al. [4] reported that adverse events occurred in 19,726 (8.6%) of 229,230 patients treated with acupuncture, and adverse events requiring additional treatment occurred in 4,963 (2.2%) patients. Although acupuncture is a relatively safe treatment, the occurrence of fatally serious adverse events should be recognized.

In this case, a patient with no medical history developed a hemothorax after acupuncture treatment. Since spontaneous or menstrual-associated pneumothorax was ruled out on the basis of intraoperative findings, the hemothorax was thought to be caused by an acupuncture-associated injury to the left second intercostal artery. Acupuncture complications are assumed to be caused by improper insertion or manipulation of needles [5]. In this case, a deeply inserted acupuncture needle likely caused direct injury to the second intercostal artery.

TAE is known to be a minimally invasive and highly successful treatment for active bleeding, and a high hemostatic success rate of 87% has been reported for bleeding from the intercostal artery [6]. To the best of our knowledge, there are no reports of TAE treatment for a hemothorax caused by acupuncture. In the present case, hemostasis was successfully achieved with TAE, without complications. An acupuncture-induced hemothorax is a rare but severe complication that requires therapeutic intervention, and TAE may be a favorable treatment option for such a complication.

## Patient consent statement

Written informed consent for publication was obtained from the patient.
